# Healthy Penis: San Francisco's Social Marketing Campaign to Increase Syphilis Testing among Gay and Bisexual Men

**DOI:** 10.1371/journal.pmed.0030474

**Published:** 2006-12-26

**Authors:** Katherine Ahrens, Charlotte K Kent, Jorge A Montoya, Harlan Rotblatt, Jacque McCright, Peter Kerndt, Jeffrey D Klausner

## Abstract

The authors describe the development, implementation, and evaluation of their innovative social marketing campaign.

## The Problem: Sharp Increase in Syphilis among Men Who Have Sex with Men

**Figure 1:**
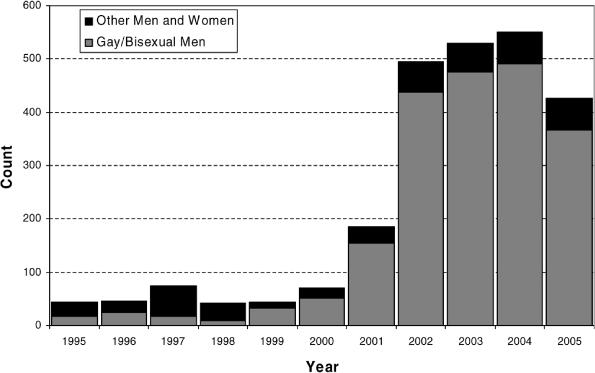
Proportion of Early Syphilis Cases among Gay/Bisexual Men in San Francisco, 1995–2005

San Francisco experienced a sharp rise in early syphilis between 1999 and 2002, with the number of cases rising from 44 to 494 per year (Figure 1). Rates continued to rise through 2004. Since 1999, most syphilis cases have been among men who identified as gay or bisexual (88%), were white (60%), and were infected with HIV (61%) (Figure 1). In June 2002, the San Francisco Department of Public Health (SFDPH), STD Prevention and Control Services launched a social marketing campaign, called Healthy Penis, designed to increase syphilis testing and awareness among gay and bisexual men.

Social marketing, like marketing in the private sector, is a research-driven approach to behavior change [1]. Unlike commercial marketing, however, it is truly consumer-centered and designed to increase the health of the consumer [2]. The success of any social marketing campaign can be attributed to the net effect of its five main components: branding, segmentation, price, placement, and promotion [1–3]. The first component, *branding*, focuses on the health behavior message so that the desired behavior, or product, appeals to the needs and values of the consumer through functional and emotional attributes.

The second component is concerned with the *segmentation* of the intended audience—developing a campaign message that emphasizes the target population's values, attitudes, and beliefs and/or capitalizes on their current stage of behavior change. Messages that are customized in this manner will ensure that the health behavior (product) is appealing and applicable to each subgroup.

The third component is *price*. In social marketing, price is the social, psychological, or physical cost the consumer associates with performing the health behavior. The fourth component is product *placement*. In public health, this involves delivering the resources that make the desired health behavior possible at a time when it will most likely be sought out. The fifth component is the *promotion* of the health behavior through communication media like print, television, radio, outdoor advertising, or face-to-face techniques that are determined by the information-consumption habits of the consumer. Notable successful social marketing campaigns include North Carolina's “Click It or Ticket” campaign aimed at increasing seatbelt use, Florida's “truth” anti-smoking campaign, and the United States National WIC (Women, Infants and Children) Breastfeeding Promotion project [4].

The impact of a social marketing campaign is evaluated based on the measurement of change in the targeted behavior, i.e., increased syphilis testing in the case of Healthy Penis, and the extent to which this change is associated with people who received the campaign messages. To that end, we initially evaluated the Healthy Penis campaign in 2002–2003 and found a higher rate of syphilis testing among those aware of the campaign than those unaware of the campaign [5]. In addition, syphilis knowledge was higher among those aware, a secondary goal of the campaign.

In this article we describe the development, implementation, and evaluations of the Healthy Penis campaign. We present a simple method to evaluate the immediate and long-term effectiveness of a public health media campaign designed to change behavior. We include our most recent evaluation, conducted in 2004–2005, which assessed the long-term effectiveness of the campaign [6] and determined if respondents aware of the campaign 2.5 years after it began displayed the same syphilis knowledge and testing behavior as those initially aware of the campaign in the first evaluation.

## Healthy Penis Campaign Development

The Healthy Penis campaign was launched during the annual San Francisco Lesbian Gay Bisexual Transgender Parade in late June 2002 and continued through December 2005. A San Francisco–based social marketing firm, Better World Advertising, created the campaign in collaboration with the SFDPH to target the gay and bisexual community. This campaign was part of a broad multifaceted effort begun in 1999 by the SFDPH in response to the syphilis epidemic [7].

The campaign was developed in collaboration with the Los Angeles–based Stop the Sores syphilis prevention campaign, which allowed us to lower our start-up costs to $75,000 in 2002. The campaign was continued through the end of 2005 at an additional cost of $295,000 [7]. Three-quarters of the campaign's first-year funds were spent on campaign development, with the remaining quarter spent on displaying campaign materials [8].

Syphilis is often initially asymptomatic and testing is readily available in San Francisco for easy diagnosis. Based on these characteristics and input from the community partners group, the SFDPH determined that the primary campaign health behavior message should be “get tested,” with the main objective of changing community norms around syphilis testing. Secondary objectives were to increase awareness about the syphilis epidemic in gay and bisexual men and to increase knowledge about syphilis (symptoms, routes of transmission, the link between syphilis and HIV transmission, and so on). The complete methodology for selection of campaign concepts and themes for this campaign has been described in a previous manuscript [5].

**Figure 2:**
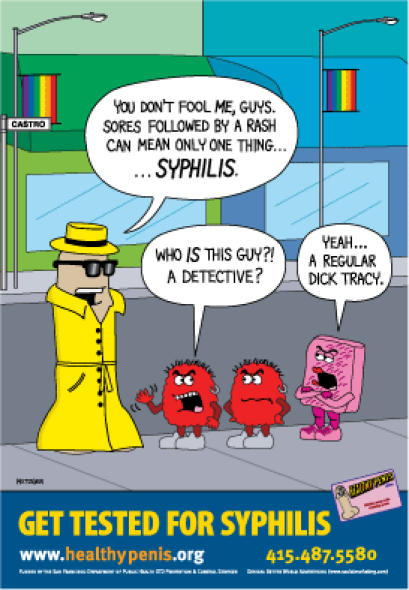
Example of Healthy Penis Campaign

The Healthy Penis campaign was promoted in neighborhoods where the greatest concentration of gay or bisexual men lived and where there were businesses that catered to this population. The campaign incorporated the use of humorous cartoon strips that featured characters like Healthy Penis and Phil the Sore to: (1) promote syphilis testing, (2) publicize the rise of syphilis among gay and bisexual men, (3) provide information on syphilis transmission, symptoms, and prevention, and (4) delineate the connection between syphilis and HIV (Figure 2). These cartoon strips were initially published semi-monthly in a popular gay Bay Area publication. After publication, poster-size reproductions were posted on the streets; in bars and commercial sex venues; on bus shelters and bus advertising; on palm cards; and on banner advertisements on one of the most popular Internet sites for meeting sex partners among gay men.

Topics addressed in campaign cartoons initially emphasized all four areas described above and then after 1.5 years focused primarily on the message “get tested.” In addition to messages contained within the cartoon strip, brief text boxes were displayed on the bottom of the cartoon images describing modes of syphilis transmission (skin-to-skin contact, oral, anal, and vaginal sex), symptoms (painless sore and/or rash), and explaining that syphilis is curable. These text boxes were displayed on cartoons from June 2002 to October 2003 with decreasing frequency.

## Evaluation of the Campaign: What Impact Has It Had?

### Survey methodology.

To evaluate the effectiveness of the campaign, we conducted two waves of surveys using the same instrument: one six months after the campaign began (December 2002–February 2003; Evaluation I), and a second 2.5 years after the campaign began (September 2004–March 2005; Evaluation II). For each survey, men were intercepted at coffee shops, bars, markets, laundromats, sex clubs, a clean and sober community center, on sidewalks, and in other venues located in campaign-targeted neighborhoods.

Respondents were asked about basic demographic information; unaided (e.g., spontaneous mention) and aided (e.g., prompted response) awareness of the Healthy Penis campaign; perceived key messages of the campaign; syphilis knowledge (via open-ended questions); sexual practices in the past month; HIV status; and how many times they were tested for syphilis in the past six months.

Comparisons were made among those aware of the campaign between the first and second sample of respondents. Recent history of syphilis testing was compared with campaign awareness level for each evaluation separately. Analysis included Fisher's Exact and Cochran-Armitage tests, and logistic regression.

### Survey population.

Two hundred and forty-four interviews were conducted with San Francisco residents and included in the first evaluation; 150 interviews were included in the second evaluation. For each evaluation, all respondents were men who have sex with men (MSM) and most were white and HIV negative. Our respondents were similar to the MSM population in San Francisco. More detailed characteristics of the two samples are described in Table 1. There were no significant demographic differences between Evaluation I and II participants.

**Table 1:**
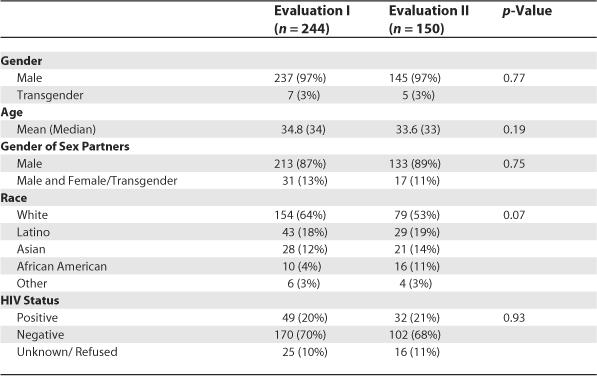
Demographic Characteristics of Respondents from First and Second Evaluations of the Healthy Penis Campaign

### Campaign awareness.

Campaign awareness was high with 80% (*n* = 194/244) and 85% (*n* = 127/150) of respondents aware of the campaign in Evaluation I and II, respectively. Unaided and aided awareness was similar between the two evaluations; 33% versus 41% spontaneously mentioned the Healthy Penis campaign (unaided awareness) when asked to recall recent advertisements or public events about sexual health issues, and an additional 47% versus 44% recognized the campaign (aided awareness) when shown a campaign image (Evaluation I and II, respectively). There was no difference in overall campaign awareness, unaided awareness, or aided awareness between the two evaluations (*p* = 0.23, *p* = 0.13, *p* = 0.60, respectively). Among respondents aware of the campaign, campaign exposure was greater in the first evaluation; Evaluation I respondents reported a median of six exposures (mean, 18) over the previous three months, while Evaluation II respondents reported a median of five times (mean, six) in the previous three months (Wilcoxon two-sample test, *p* < 0.0001). Perceptions of key messages among respondents who were aware of the campaign did not change markedly between Evaluations I and II, with the most common message identified being “get tested” (53% versus 55%, respectively, *p* = 0.73).

### Syphilis testing in the last 6 months.

**Figure 4:**
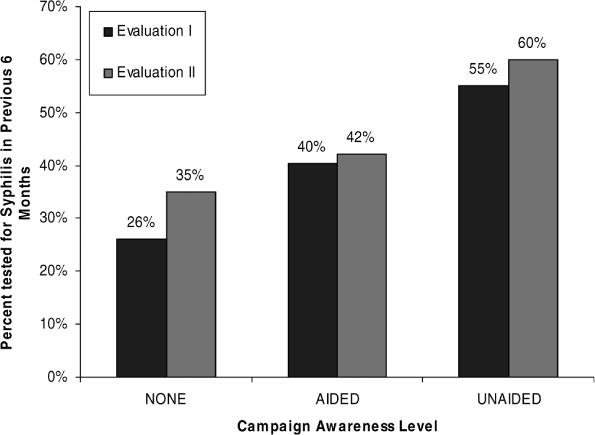
Syphilis Testing by Healthy Penis Campaign Awareness Level and Evaluation Period

Both evaluations found a positive association between campaign awareness and recent syphilis testing—the primary objective of the social marketing campaign. An increasing proportion of respondents reported syphilis testing in the previous six months by campaign awareness level (none, aided awareness, and unaided awareness): Evaluation I: 26%, 40%, 55% (Cochran-Armitage trend test z = −3.303, *p* = 0.001); Evaluation II: 35%, 42%, 60% (Cochran-Armitage trend test z = −2.304, *p* = 0.02) (Figure 4). After controlling for potential confounders (age, HIV status, casual sex partners in the last month) in a multivariable logistic regression model, each increase in campaign awareness level during Evaluation I was associated with a 90% increase in likelihood for having tested for syphilis in the past six months (odds ratio [OR] 1.9 [95% confidence interval (CI), 1.3–2.9]). Other variables significantly related to syphilis testing in Evaluation I included being HIV infected (OR 4.9 [95% CI, 2.3–10.5]), and having casual sex partners in the last month (OR 2.0 [95% CI, 1.1–3.9]). The same multivariable model applied to Evaluation II respondents found each increase in campaign awareness level to be associated with a 76% increase in likelihood for syphilis testing (OR 1.76 [95% CI, 1.01–3.1]), and no other factors were significant in the model, although HIV-infected respondents showed an increased likelihood of syphilis testing (OR 2.1 [95% CI, 0.9–5.2], *p* = 0.10). In both evaluations, a higher proportion of HIV-positive respondents reported syphilis testing than did HIV-negative respondents (Evaluation I 71% versus 34%, p < 0.05, Evaluation II 63% versus 44%, *p* = 0.07).

Overall, reported syphilis testing in the previous six months did not change from the first evaluation to the second evaluation (42% versus 49%, *p* = 0.25).

### Syphilis knowledge.

**Figure 3:**
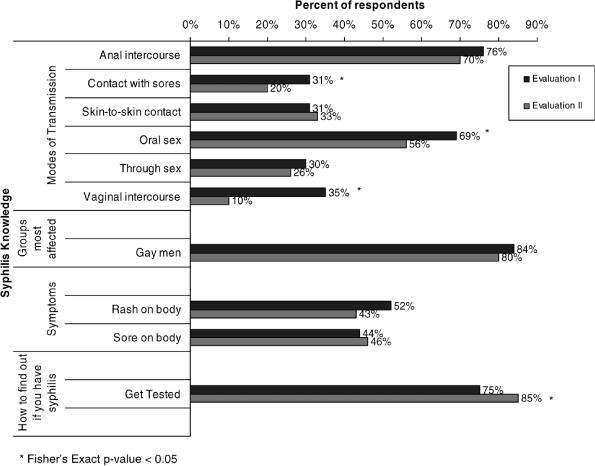
Syphilis Knowledge among Respondents Aware of the Healthy Penis Campaign, by Evaluation Period

There were few significant differences between evaluations among respondents aware of the campaign in terms of knowledge of syphilis, including knowledge of symptoms; groups most affected by syphilis; how to find out if you have syphilis; health consequences of untreated syphilis; and the relationship between syphilis and HIV acquisition and transmission (Figure 3).

### Syphilis incidence.

In 2005, incidence of early syphilis was lower than in the previous three years, with decreases in cases in gay/bisexual men accounting for the drop [9]. Although our campaign evaluations were cross-sectional and causality cannot be determined, it does appear that the Healthy Penis campaign, along with other SFDPH syphilis elimination efforts [7], may have led to this decrease in syphilis incidence.

## Conclusions from Evaluations

MSM who were aware of the Healthy Penis campaign were more likely than those unaware to have recently tested for syphilis and to have greater knowledge about syphilis. This effect was sustained for almost three years.

## Next Steps for Healthy Penis

After initial evaluation of the Healthy Penis campaign, elements have been used in Philadelphia, Seattle, Palm Springs, and Santa Clara County, California (www.healthypenis.org). Thus the campaign can be translated to other jurisdictions. Because much of the material is already developed, other jurisdictions may adapt and use the campaign at a lower cost than was originally incurred in San Francisco, although they will need to conduct evaluations to ensure the campaign is effective for their population. In addition, the principles used in the development, implementation, and evaluation of Healthy Penis are universally applicable for any social marketing campaign.

Based on our experience with the Healthy Penis campaign, we believe spending more start-up resources on campaign development rather than campaign placement [8] was probably what led to our high level of campaign awareness, demonstrating that our campaign resonated with our target population. We also learned that partnering with another jurisdiction (Los Angeles) lowered start-up costs and creating a Community Partners Group helped us tailor our campaign messages to be most effective. And most importantly, we learned that careful, well-planned evaluations are critical in assessing the impact of a campaign, which then supported continuing the campaign and implementing it elsewhere. Our evaluations strongly suggest that the Healthy Penis social marketing campaign was effective in augmenting syphilis testing and increasing syphilis awareness and knowledge in the San Francisco gay and bisexual community. This effect might have contributed to decreased syphilis incidence in 2005.

## Abbreviations

CI: confidence interval
MSM: men who have sex with men
OR: odds ratio
SFDPH: San Francisco Department of Public Health

## References

[pmed-0030474-b001] Lamptey PR, Price JE (1998). Social marketing sexually transmitted disease and HIV prevention: A consumer-centered approach to achieving behaviour change. AIDS.

[pmed-0030474-b002] Andreasen AR (1995). Marketing social change: Changing behavior to promote health, social development, and the environment.

[pmed-0030474-b003] Aaker DA (1996). Building strong brands.

[pmed-0030474-b004] Social Marketing Institute (2000). Success stories. http://www.social-marketing.org/success.html.

[pmed-0030474-b005] Montoya JA, Kent CK, Rotblatt H, McCright J, Kerndt PR (2005). Social marketing campaign significantly associated with increases in syphilis testing among gay and bisexual men in San Francisco. Sex Transm Dis.

[pmed-0030474-b006] Ahrens KA, Kent CK, Montoya JA, Rotblatt H, McCright J (2006). Healthy Penis social marketing campaign associated with increased syphilis testing for three years in San Francisco [poster].

[pmed-0030474-b007] Klausner JD, Kent CK, Wong W, McCright J, Katz MH (2005). The public health response to epidemic syphilis, San Francisco, 1999–2004. Sex Transm Dis.

[pmed-0030474-b008] Vega M, Roland E (2005). Social marketing techniques for public health communication: A review of syphilis awareness campaigns in 8 US cities. Sex Transm Dis.

[pmed-0030474-b009] Klausner JD, Kent CK, Kohn RP, Nieri G, McCright J The changing epidemiology of syphilis and trends in sexual risk behavior, San Francisco, 1999–2005 [poster].

